# Hematoma Interleukin‐1 Receptor Antagonist Concentrations Predict Long‐Term Outcome in Acute Human Intracerebral Hemorrhage

**DOI:** 10.1002/ana.78237

**Published:** 2026-05-03

**Authors:** Adrian R. Parry‐Jones, Blessing Nyakutsikwa, Michael H. Askenase, Matthew Gittins, Mary Newland, Siobhan Crilly, Paul R. Kasher, Yvonne Davidson, Federico Roncaroli, Stuart M. Allan, Lauren H. Sansing, Wendy Ziai, Daniel F. Hanley, INFLAME‐ICH Investigators, Mark Harrigan, David Miller, Judy Huang, Wendy Ziai, Jack Jallo, Fred Rincon, Gaurav Gupta, Igor Rybinnik, Diederik Bulters, Mary Leigh Gelea, Andras Buki, Erzsebet Ezer, Hiren Patel, Adrian Parry‐Jones, Charles Matouk, Kevin Sheth / Lauren Sansing, Ali Zomorodi, Michael James, Pal Barzo, Krisztian Tanczos, Gregory Thompson, Ventatakrishna Rajajee/Aditya Pandey, Peter Nakaji, Shawn E. Wright, Walter Galicich, Thomas Bergman, Andrew Carlson, Huy Tran, Philipp Taussky, Safdar Ansari, Fuat Arikan, Marcelino Baguena, Ronald Reimer, W. David Freeman, Ania Pollack, John Terry, Geza Mezey, Katalin Szabo/Laszlo Csiba, Robert F. James, Steven J. Barrer, Larami MacKenzie

**Affiliations:** ^1^ Division of Cardiovascular Sciences, Faculty of Biology, Medicine and Health University of Manchester Manchester UK; ^2^ Geoffrey Jefferson Brain Research Centre, Manchester Academic Health Science Centre, Northern Care Alliance NHS Foundation Trust & University of Manchester Manchester UK; ^3^ Centre for Biostatistics, Faculty of Biology, Medicine and Health, University of Manchester Manchester UK; ^4^ Department of Immunobiology Yale University School of Medicine New Haven CT; ^5^ Division of Neuroscience Faculty of Biology, Medicine and Health, University of Manchester Manchester UK; ^6^ Department of Neurology Yale University School of Medicine New Haven CT; ^7^ Department of Anesthesiology and Critical Care Medicine Johns Hopkins University School of Medicine Baltimore MD; ^8^ Department of Neurology Johns Hopkins University School of Medicine Baltimore MD

## Abstract

**Objectives:**

The interleukin (IL)‐1, IL‐6, and C‐reactive protein (CRP) pathway is central to the immune response after intracerebral hemorrhage (ICH). We tested for associations between hematoma and plasma cytokine concentrations and patient outcomes in Minimally Invasive Surgery Plus Rt‐PA for ICH Evacuation Phase III (MISTIE III) participants.

**Methods:**

Inflammation after minimally invasive evacuation of ICH (INFLAME)‐ICH was a sub‐study nested in MISTIE III. Daily hematoma fluid was collected from surgical patients and peripheral blood for all patients. Multiple regression models compared hematoma cytokine concentrations to the modified Rankin scale (mRS) score at 1 year. Correlations between hematoma and plasma cytokine concentrations were tested. We compared plasma cytokines in patients randomized to surgery (vs medical). Gene expression in monocyte/macrophages and neutrophils were compared in a subset of participants.

**Results:**

A total of 89 patients were recruited (47 surgical, 42 medical). Mean hematoma IL‐1 receptor antagonist (IL‐1Ra) (odds ratio [OR]: 5.92; 95% confidence interval [CI]: 1.08–32.54; n = 38) and mean hematoma IL‐6 (OR: 3.23; 95% CI: 1.33–7.81; n = 45) were independently associated with good outcome (mRS, 0–3) at 1 year. Higher hematoma IL‐1β was associated with higher plasma CRP (β‐coefficient: 21.0; 95% CI: 4.4–37.5; *n* = 117 paired samples). No differences were seen in plasma IL‐6, CRP and IL‐1Ra in patients by treatment group. *IL1B*, *IL6*, and *IL1RN* transcripts in monocyte/macrophages correlated with respective protein concentrations in hematoma fluid.

**Interpretation:**

Hematomal IL‐1Ra within a week of ICH is independently associated with a good outcome at 1 year, supporting further investigation of IL‐1Ra in ICH. IL‐6 is independently associated with a good outcome at 1 year, which might suggest a role in enhancing repair and recovery. ANN NEUROL 2026;100:345–360

Intracerebral hemorrhage (ICH) has a 1‐month case fatality rate of 30 to 40% and only 1 in 5 patients regain independence.^1^ Globally, hemorrhagic stroke accounts for 45.6% of 7.3 million annual stroke deaths and 50% of disability adjusted life years lost to stroke.^2^ Supportive care, anticoagulant reversal, and intensive blood pressure lowering are recommended^3^ and minimally invasive surgery shows promise.^4^, ^5^ Reducing perihematomal secondary brain injury may benefit the great majority of ICH patients as a stand‐alone treatment in smaller hematomas and as an adjunct to surgery for large bleeds.^6^

Preclinical studies have demonstrated that local inflammation occurs in response to diverse acute brain injuries, exacerbating early damage, but also playing an important role in later repair and recovery.^7^ The pro‐inflammatory cytokine interleukin (IL)‐1 plays a key detrimental role and inhibiting IL‐1 leads to a reduction in damage in diverse experimental acute brain injuries.^8^ IL‐1 is rapidly upregulated in perihematomal brain tissue from animal models of ICH^9^ and from humans.^10^ IL‐1 receptor antagonist (IL‐1Ra) is a naturally occurring IL‐1 blocker and upregulation of endogenous IL‐1Ra leads to a reduction in edema and neutrophil infiltration in an animal model of ICH.^11^ However, studies in ischemic stroke show that findings in preclinical studies may not translate directly to patients.^12^ There is, therefore, a need to use novel studies in clinical ICH to bridge this translational gap.^12^

Previous clinical studies in ICH have measured peripheral inflammatory markers on admission and shown associations with worse short term outcomes,^13^, ^14^, ^15^, ^16^, ^17^ as well as with poor functional outcomes and survival at 1 to 3 months.^14^, ^17^, ^18^ Postmortem studies have shown inflammatory cell infiltration in the brain around the hemorrhage,^19^ but we know very little about the nature and temporal profile of the inflammatory response in clinical ICH and how it relates to systemic inflammation. The Minimally Invasive Surgery Plus Rt‐PA for ICH Evacuation Phase III (MISTIE III) trial tested minimally invasive catheter evacuation followed by thrombolysis against standard care.^20^ The trial provided a unique opportunity to investigate key aspects of the inflammatory response directly within the brain in humans. We measured the main cytokines of the IL‐1 family (IL‐1α, IL‐1β, and IL‐1Ra) and IL‐6, a key downstream mediator of IL‐1.^21^ We first sought to test whether these cytokines were detectable in the hematoma fluid of MISTIE III patients randomized to surgery and if so, aimed to describe their temporal profile. Perihematomal edema (PHE) and blood brain‐barrier breakdown^22^, ^23^ is known to differ by location, so we tested whether cytokine concentrations were associated with bleed location (deep vs lobar). We then tested whether cytokine concentrations in the hematoma were associated with subacute PHE and outcome at 1 year. Next, we investigated expression of the genes encoding these cytokines in key myeloid cell populations isolated from hematoma fluid and peripheral blood in a subset of surgical patients. We, then, sought to determine whether hematoma cytokine concentrations (IL‐1, IL‐6, and IL‐1Ra) were associated with plasma concentrations of IL‐6, C‐reactive protein (CRP), and IL‐1Ra. Finally, we tested whether the MISTIE procedure caused a significant increase in circulating IL‐6, CRP, and IL‐1Ra when compared to those randomized to standard care.

## Materials and Methods

Inflammation after minimally invasive evacuation of ICH (INFLAME‐ICH) was a prospective observational sub‐study nested in the MISTIE III (NCT01827046) clinical trial. INFLAME‐ICH was conducted at 27 MISTIE III centers (United States [US] = 15, United Kingdom [UK] = 4, Spain = 5, Hungary = 3). Ethical approval at each site was secured through an amendment to the main MISTIE III ethics approval, which was approved by institutional review boards for each center. INFLAME‐ICH was presented as an optional sub‐study and participants willing to take part in INFLAME‐ICH signed an additional consent form, separate from their consent for the main MISTIE III trial.

### MISTIE III Procedures

The MISTIE III trial is described in detail elsewhere.^20^ Patients with spontaneous supratentorial ICH with a hematoma volume of 30mL or more were included. Symptom onset to diagnostic computed tomography (CT) was less than 24 hours, and randomization had to be performed between 12 and 72 hours after the diagnostic CT. Patients randomized to the MISTIE intervention had a rigid cannula placed within the hematoma through a burr hole or twist drill opening. Clot aspiration was performed with a handheld syringe until first resistance. A soft catheter was placed into the residual hematoma and connected to a 3‐way stopcock and closed drainage system. Six hours or more after catheter placement, alteplase (1mg in 1mL) was administered directly into the clot through the catheter every 8 hours, for up to 9 doses. Alteplase was stopped when the surgical aim (residual hematoma ≤15mL) was reached, 9 doses of alteplase were given, or a clinically symptomatic rebleeding event occurred. All doses were followed by a 3mL flush of preservative‐free normal saline, and the system was closed for 1 hour to allow drug–clot interaction and then reopened to allow for gravitational drainage. The diagnostic CT scan and the CT scan closest to 120 hours (5 days) after symptom onset were used for analysis of PHE. Day 5 CT scans were a median of 117.3 hours (interquartile range [IQR]: 112.0–123.6 hours, range: 65.7–142.5 hours) after symptom onset. CT scans were analyzed by the imaging center at Johns Hopkins University, Baltimore, MD. Digital imaging and communications in medicine images were loaded into OsiriX MD (version 9.0.1) to determine hematoma and PHE volume and hematoma location (lobar vs deep).^24^

### INFLAME‐ICH Sample Collection and Processing

INFLAME‐ICH participants randomized to surgery had hematoma aspirate collected at the time the drain was placed surgically. This was the liquid component of the hematoma (if any) that the surgeon was able to aspirate before drain placement. This sample was collected before the introduction of alteplase. A hematoma fluid sample was then collected every subsequent day that the drain remained in situ. Alteplase was administered directly into the clot through the drain, at 1.0mg in 1mL followed by 3mL flush every 8 hours, for up to 9 doses. The duration for which the drain remained in situ was determined by the main trial protocol. Once the surgical goal had been achieved, the drain was removed. The goal was reached if the residual hematoma was ≤15mL, 9 doses of alteplase were given, or on occurrence of a clinically symptomatic rebleeding event. Daily peripheral blood was collected at the time of hematoma aspirate or hematoma fluid collection. Patients randomized to standard medical care had daily peripheral blood collection, starting on the day of randomization and at 09:00 ± 2 hours for 4 further days.

Hematoma aspirate was immediately transferred to an additive‐free plastic collection tube. For collection of hematoma fluid after drain insertion, the drip chamber in the system was emptied, then hematoma fluid accumulated over a 5‐hour interval before collection, before the alteplase dose scheduled between 08:00 and 16:00. This sample was collected during the final 5 hours of an 8‐hour interval since the previous dose of alteplase. On the final collection day only, and once the drain had been removed, the contents of the tubing between the drain and the drip chamber were collected. All peripheral blood was collected using 6mL EDTA tubes. Samples were centrifuged (<1 hour after collection) at 2,000*g*, at room temperature, for 10 minutes. Supernatant was transferred into cryovials and frozen at −70°C or colder. All samples were shipped on dry ice to the central laboratory at the Biomedical Facility, Salford Royal Hospital, Salford, UK.

INFLAME‐ICH patients in the United States were invited to contribute samples to the ICH‐Seq study, as previously reported.^25^ Samples were collected for ICH‐Seq at the same time points as INFLAME‐ICH and transferred to a central laboratory for processing. Leukocytes were isolated from hematoma fluid and peripheral blood, and myeloid populations were separated using fluorescence‐activated cell sorting. Transcriptomic profiling of CD14+ monocytes/macrophages and CD66a/c/e + neutrophils was performed and data describing expression of the *IL‐1A*, *IL‐1B*, *IL‐6*, and *IL‐1RN* genes were analyzed.

### Sample Assays

IL‐6 (hematoma and plasma), IL‐1β (hematoma), IL‐1α (hematoma), and CRP (plasma) were analyzed using Luminex technology (Luminex, Austin, TX). Bio‐Plex magnetic COOH beads (Bio‐Rad Laboratories, Hemel Hempstead, UK) were coupled to specific monoclonal antibodies (PeliKine anti‐IL‐6, cat. M191602; PeliKine anti‐IL‐1β, cat. M193402; IL‐1α R&D DuoSet, cat. 840201; Biodesign anti‐CRP, cat. M86842M; clone C2, Amsbio, Abingdon, UK). The analytes were secondarily bound with specific antibodies conjugated to biotin (PeliKine anti‐IL‐6, cat. M191604; PeliKine anti‐IL‐1β, cat. M193404; IL‐1α R&D DuoSet, cat. 840202), except for CRP, which was measured in a single‐plex competitive assay, where the competitor used was SCIPAC CRP cat. P100‐0 (SCIPAC, Sittingbourne, UK) biotinylated with Pierce EZ‐Link Sulfo‐NHS‐LC–LC‐Biotin (Pierce, Rockford, IL). Following addition of Jackson Laboratories R‐Phycoerythrin Streptavidin (cat. 016‐110‐084; Stratech, Newmarket, UK) signal was read using a Bio‐Plex 200 system. IL‐1Ra and IL‐6 were analyzed using enzyme‐linked immunosorbent assay (ELISA). Specific monoclonal antibodies were captured on Costar 96‐well microplates (cat. 3,590; Appletonwoods, Kings Norton, UK), Peprotech anti‐IL‐1Ra (cat. 900‐K474; Peprotech EC), PeliKine anti‐IL‐6 (cat. M191602), and secondarily bound with Invitrogen biotinylated mouse monoclonal anti‐IL‐1Ra (cat. AHC0219) clone A71B 6D11 (Life Technologies, Paisley, UK), and PeliKine anti‐IL‐6 (cat. M191604). Following addition of Jackson Laboratories streptavidin‐HRP (cat. 016‐030‐084; Stratech, Newmarket, UK), signal was read using a Wallac Victor 1,420 multilabel counter. All cytokine measurements were standardized relative to the corresponding National Institute for Biological Standards Control (NIBSC, South Mimms, UK). All biomarker measurements were performed blinded to clinical and neuroimaging findings.

### Postmortem Brain Analysis

To understand whether IL‐1Ra expression in the tissue around the hematoma might have contributed to the IL‐1Ra detected in the hematoma fluid, we examined 9 postmortem brains. Methods and results are provided in the Supporting Information.

### Statistical Analysis

Cytokine concentrations were log transformed for all graphs and analyses. When plotting over time, a line of best fit and 95% confidence interval (CI) was derived using natural cubic splines. Good outcome was defined as a modified Rankin scale (mRS) score at 1 year of 0 to 3 (vs poor outcome of 4–6). This definition was chosen as it is the primary outcome of the main MISTIE III trial, mRS 0 to 3 represents a good outcome for the severe ICH patients included in MISTIE III, and because we were primarily interested in the effect of the inflammatory response on long‐term outcome. For each participant, the mean of cytokine concentrations was calculated and used to test for association with mRS. We adjusted for age, time from onset to surgery, and severity index, as previously described.^20^ A previous analysis of ICH‐Seq suggested 2 distinct stages of gene expression in hematoma myeloid cells. An acute stage spanning the first 4 days post‐ICH, during which myeloid transcriptional profiles were rapidly changing, followed by a sub‐acute stage spanning 4 to 7 days post‐ICH, during which they were more stable.^25^ We, therefore, investigated whether determining mean cytokine concentrations before or after a fixed time point improved model fit, compared with the mean of all time points combined. We split cytokine data into those collected before 72 hours post‐ICH and those after 72 hours, and we calculated the mean cytokine concentration for each period. Additional analyses using time cut points every 12 hours after 72 hours, up to 120 hours, were also performed. We determined the Akaike information criterion (AIC) and Bayesian information criterion (BIC) of regression models with cytokine data divided based on a fixed time point and compared these to the AIC and BIC of models where all time points were averaged together. We compared hematoma cytokine concentrations by location using multiple linear regression models, with cytokine concentration as outcome, location as the explanatory factor and adjusting for ICH volume, severity index, age, and onset to surgery time. The severity index was a score developed for the MISTIE III trial, combining multiple prognostic factors and is described in the Supporting Information methods.

We used the PHE volume at day 5 as a measure of PHE. We tested for associations between hematoma and plasma cytokine concentrations and PHE volume at day 5 using multiple linear regression and adjusted for severity index, age, and baseline PHE volume.

For ICH‐Seq participants, we used linear regression to test for associations between gene expression (*IL‐1A*, *IL‐1B*, *IL‐6*, and *IL‐1RN*) in monocyte/macrophages and neutrophils and the concentration of protein (IL‐1α, IL‐1β, IL‐6, and IL‐1Ra) in the same tissue compartment at the same time point, adjusting for time from symptom onset to sample collection.

We tested for associations between hematoma cytokine concentrations and plasma cytokine concentrations using pairs of samples taken on the same day where the plasma sample was collected at the same or later than the hematoma sample. We used multiple linear regression and included hours from onset to hematoma fluid collection, hours between hematoma fluid, and plasma sample collection, severity index, and age.

Finally, we compared the concentrations of plasma IL‐6, CRP, and IL‐1Ra for participants randomized to surgery in MISTIE III versus medical care, adjusting for severity index and age, introducing an interaction term for treatment group and time from onset in multifactorial linear regression models.

## Results

### Baseline Characteristics and Sample Collection

We recruited 89 patients, 47 surgical, and 42 medical. Baseline characteristics are in Table 1, and no significant difference was found between groups. The baseline characteristics of the MISTIE III patients included in INFLAME‐ICH are compared to those not included in INFLAME‐ICH and the whole MISTIE III cohort in Table S1. INFLAME‐ICH participants are broadly representative of the entire cohort of MISTIE III participants. Surgical patients had lower ICH volume and lower edema at day 5. Twenty (42.6%) surgical patients were also included in the ICH‐Seq study. Hematoma fluid samples were collected from surgical patients on a mean of 3.7 (standard deviation [SD]: 1.2) days and a median of 4 (IQR: 3–5; range: 1–5) days. Plasma samples were collected from surgical patients on a mean of 4.3 (SD: 1.1) days and a median of 5 (IQR: 4–5; range 1–5) days. Plasma was collected from medical patients on a mean of 4.6 (SD: 0.9) and a median of 5 (IQR: 4.25–5; range: 1–6) days. Median time to first hematoma sample was 59.7 hours, and there was no significant difference between groups for time to first plasma sample (medical 50.0 hours vs surgical 55.5 hours). Two surgical patients had hematoma fluid samples collected at 1 time point, but insufficient sample for INFLAME‐ICH assays, so were excluded from our analyses. Insufficient hematoma fluid sample was available for IL‐1Ra assays at a single time point from 7 additional patients, who were also excluded from all IL‐1Ra analyses.

**TABLE 1:** Baseline Characteristics of INLFAME Study Patients

	Surgical (n = 47)	Medical (n = 42)
Age	63 (54–68)	64 (54–71)
Sex, M	33 (70.2%)	25 (59.5%)
Race		
African American	8 (17.0%)	5 (11.9%)
Asian	2 (4.3%)	2 (4.8%)
White	37 (78.7%)	34 (81.0%)
Other	0 (0%)	1 (2.4%)
On anticoagulants	3 (6.4%)	4 (9.5%)
Diabetes	11 (23.4%)	6 (14.3%)
Hypertension	45 (95.7%)	38 (90.5%)
Glasgow Coma scale at randomization	10 (8–12)	11 (9–13)
NIHSS score at randomization	19 (15–22)	17 (14–22)
Severity index	3.4 (2.1–4.3)	3.3 (2.1–3.8)
ICH location		
Deep	27 (57.4%)	24 (57.1%)
Lobar	20 (42.6%)	18 (42.9%)
ICH volume at baseline (mL)	43.5 (33.5–54.1)	44.3 (31.9–59.0)
IVH volume at baseline (mL)	0.0 (0.0–2.7)	0.1 (0.0–2.0)
Perihematomal edema volume at baseline (mL)	28.1 (18.4–36.9)	27.3 (19.9–35.4)
ICH volume at day 5 (mL)	17.5 (7.7–24.1)	47.2 (34.9–57.0)
Perihematomal edema volume at day 5 (mL)	37.4 (26.6–50.5)	50.9 (40.8–64.3)
Ventilated at randomization	18 (38.3%)	13 (31.0%)
Symptom‐onset to first hematoma sample, h	59.7 (49.0–73.2)	N/A
Symptom‐onset to first plasma sample, h	55.5 (43.3–72.0)	50.0 (37.1–68.8)
Included in ICH‐Seq	20 (42.6%)	–
Hematoma IL‐1α (ng/mL)	2.7 (0.2–10.9)	–
Hematoma IL‐1β (ng/mL)	20.9 (5.1–44.2)	–
Hematoma IL‐1Ra (ng/mL)	3,751 (1,833–6,667)	–
Hematoma IL‐6 (ng/mL)	16574.2 (5,385.3–37852.1)	–
Plasma CRP (mg/mL)	37.3 (22.3–59.6)	27.9 (14.3–48.4)
Plasma IL‐6 (ng/mL)	27.0 (13.4–55.3)	18.8 (11.2–41.7)
Plasma IL‐1Ra (ng/mL)	462.4 (285.4–687.6)	428 (287–710)

Show as median and interquartile range, where appropriate. Median cytokine concentrations for all samples are given. *p*‐Values were derived by comparing groups using the Mann–Whitney *U* test or χ^2^ test for continuous or categorical measures, respectively. CRP = C‐reactive protein; ICH = intracerebral hemorrhage; IL = interleukin; IL‐1Ra = IL‐1 receptor antagonist; IVH = intraventricular hemorrhage; NIHSS = National Institutes of Health Stroke Scale.

### Temporal Profiles of Cytokines and Association with Location

**FIGURE 1:**
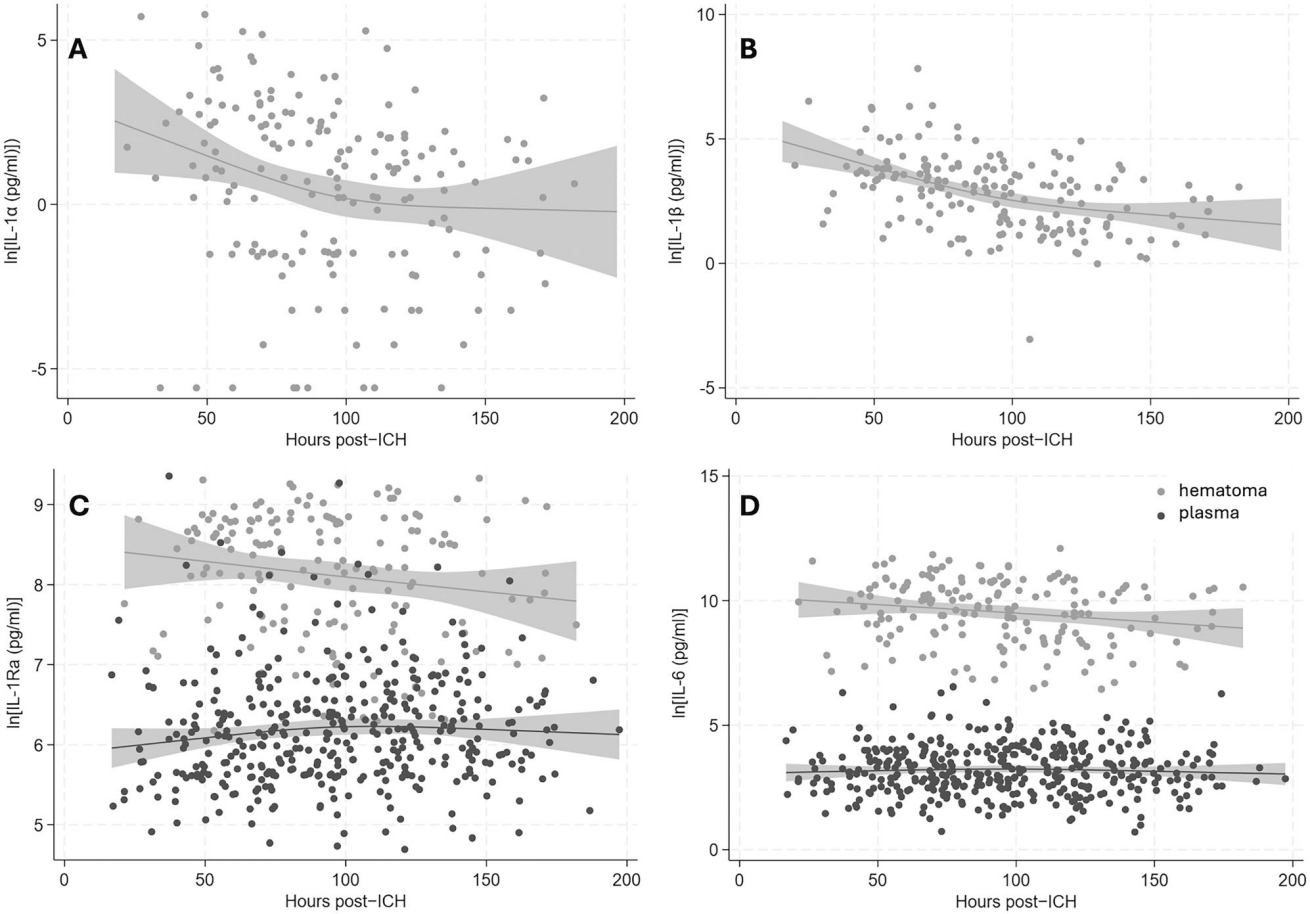
Time course of cytokines over first week. Data are shown for (A) interleukin‐1α (IL‐1α) in hematoma (light gray), (B) interleukin‐1β (IL‐1β) in hematoma, (C) interleukin‐1 receptor antagonist (IL‐1Ra) in hematoma and plasma (dark gray), (D) interleukin‐6 (IL‐6) in hematoma and plasma. The lines of best fit and 95% confidence interval are derived using natural cubic splines.

IL‐1β was detected in hematoma fluid at higher concentrations than IL‐1α and remained detectable up to the end of the sampling period (Fig 1A, B). IL‐1Ra was present in hematoma fluid at concentrations approximately 40‐fold higher than IL‐1β (Fig 1C). IL‐1Ra was detectable in the plasma at concentrations approximately 10‐fold lower than hematoma fluid. IL‐6 concentrations were high in hematoma fluid. Plasma IL‐6 was approximately 1,000‐fold lower than the hematoma fluid (Fig 1D). IL‐1Ra showed lower concentrations in lobar ICH (vs deep; β‐coefficient: −0.81; 95% CI: −1.45 to −0.16), after adjusting for baseline ICH volume, age, severity index, and onset to surgery time. No significant difference was found for IL‐1α, IL‐1β, or IL‐6 by location.

### Association between Cytokine Concentrations and Outcome

**FIGURE 2:**
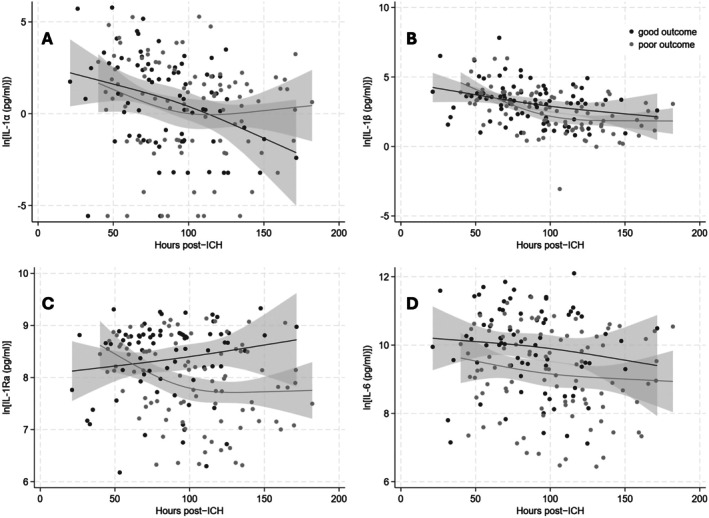
Hematoma cytokine profile over time stratified by outcome at 12 months. Good outcome (dark gray) was defined as modified Rankin scale (mRS) score of 0–3 at 12 months. Poor outcome (light gray) is defined as mRS score of 4–6 at 12 months. Profiles are shown for (A) interleukin‐1α (IL‐1α), (B) interleukin‐1β (IL‐1β), (C) interleukin‐1 receptor antagonist (IL‐1Ra), and (D) interleukin‐6 (IL‐6). The lines of best fit and 95% confidence interval are derived using natural cubic splines.

We tested for associations between hematoma cytokine concentrations and good outcome at 1 year and found no evidence for an improved model fit by applying a time cut point (Table S2). We, therefore, calculated the mean cytokine concentration for all time points from each participant and used this mean as a covariable in our regression models. After adjusting for severity index, age, and time from onset to surgery we found that IL‐1Ra (OR: 5.92; 95% CI: 1.08−32.54) and IL‐6 (OR: 3.23; 95% CI: 1.33–7.81) in the hematoma were significantly associated with better outcome, whereas the mean concentrations of IL‐1α (OR: 1.04, 95% CI: 0.70–1.54) and IL‐1β (OR: 1.41; 95% CI: 0.77–2.59) in the hematoma were not. Cytokine concentrations divided by good and poor outcome are shown in Figure 2.

### Association between Cytokine Concentrations and PHE

**FIGURE 3:**
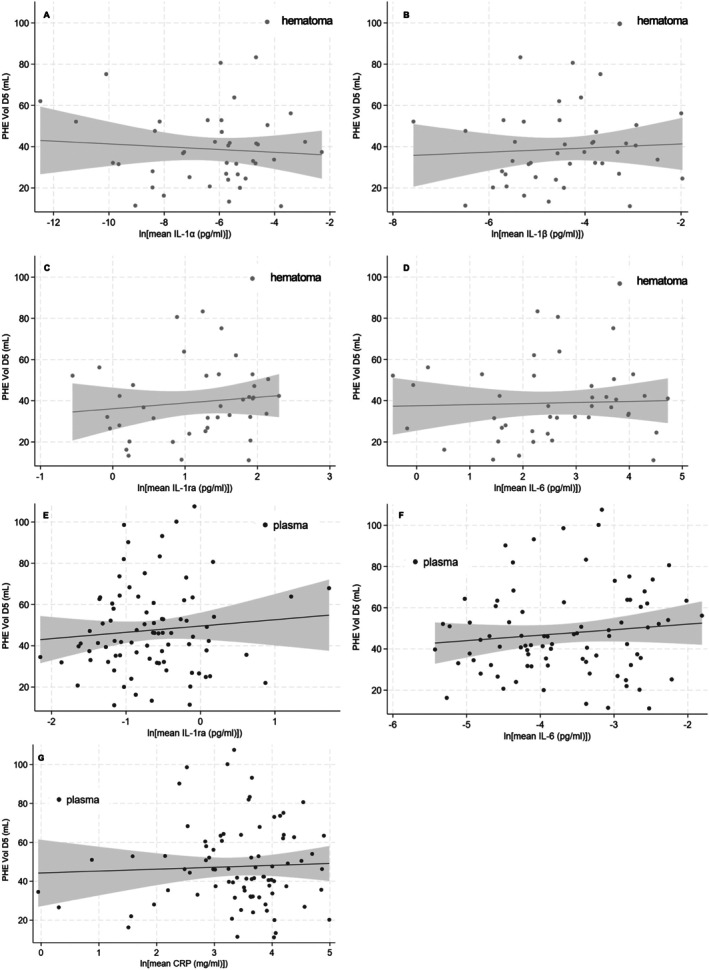
Correlation between mean cytokine concentrations and perihematomal edema at day 5. Edema is shown as the perihematomal edema (PHE) volume 5 days after symptom onset. Scatter plots are shown for (A) hematoma (light gray) interleukin‐1α (IL‐1α), (B) hematoma interleukin‐1β (IL‐1β), (C) hematoma interleukin‐1 receptor antagonist (IL‐1Ra), and (D) hematoma interleukin‐6 (IL‐6). (E) Plasma (dark gray) interleukin‐6 (IL‐6), (F) plasma interleukin‐1 receptor antagonist (IL‐1Ra), (G) plasma C‐reactive protein (CRP). Each point is the mean of concentrations at all time points for each patient. Lines were derived by simple linear regression. After adjusting for baseline PHE volume, severity index and age, no cytokine was significantly associated with PHE volume on day 5.

The relationship between mean hematoma and plasma cytokine concentrations and the PHE volume at day 5 is demonstrated in Figure 3. Multifactorial linear regression models demonstrated no significant association for hematoma IL‐1α (β‐coefficient: 1.48; 95% CI: −1.24 to 4.19), hematoma IL‐1β (β‐coefficient: 1.18; 95% CI: −2.64 to 5.00), hematoma IL‐1Ra (β‐coefficient: 7.88; 95% CI: −2.01 to 17.78), hematoma IL‐6 (β‐coefficient: 2.36; 95% CI: −2.12 to 6.85), plasma IL‐1Ra (β‐coefficient: −0.01; 95% CI: −6.04 to 6.02), plasma IL‐6 (β‐coefficient: −1.18; 95% CI: −5.85 to 3.50), or plasma CRP (β‐coefficient: −1.02; 95% CI: −5.74 to 3.71) after adjusting for severity index, age, and baseline PHE volume.

### Source of Cytokines in Hematoma Samples

**FIGURE 4:**
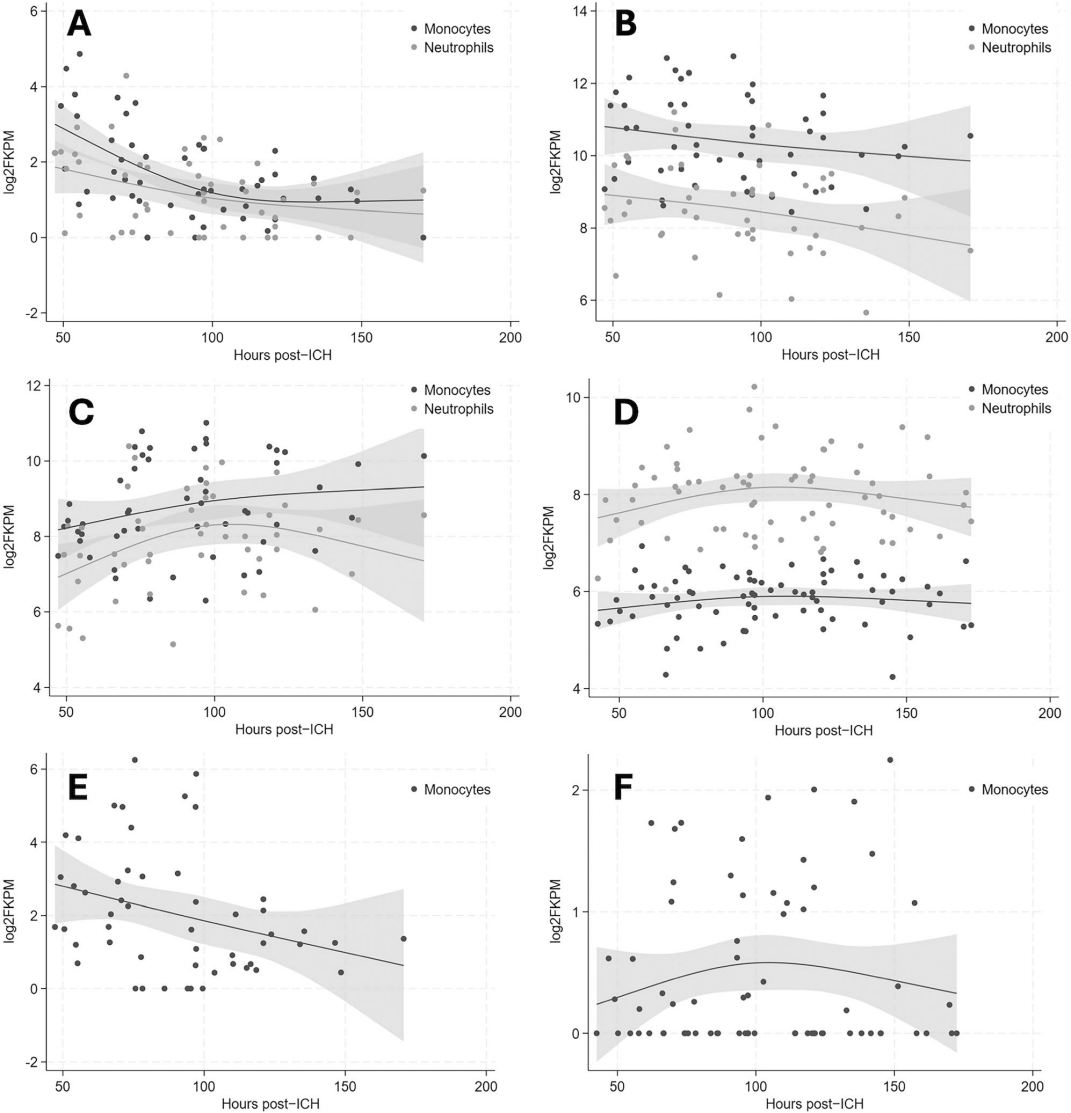
Expression of cytokines by cell type over first week. Data are shown from a subset of INFLAME patients participating in intracerebral hemorrhage (ICH)‐Seq, describing gene expression in monocyte/macrophages and neutrophils over the first week for genes encoding (A) interleukin‐1α (*IL1A*) in hematoma, (B) interleukin‐1β (*IL1B*) in hematoma, (C) interleukin‐1 receptor antagonist (*IL1RN*) in hematoma, (D) *IL1RN* in the plasma, (E) interleukin‐6 (*IL6*) in hematoma, and (F) *IL6* in the plasma. The lines of best fit and 95% confidence interval are derived using natural cubic splines. Expression in monocyte/macrophages is shown in dark gray and expression in neutrophils in light gray.

Data from ICH‐Seq were available for 20 participants in the surgical arm and expression of the genes encoding IL‐1α (*IL1A*), IL‐1β (*IL1B*), IL‐6 (*IL6*), and IL‐1Ra (*IL1RN*) are shown in monocyte/macrophages and in neutrophils in Figure 4. In hematoma fluid, *IL1A* was equally expressed in both cell types, whereas monocyte/macrophages showed a greater expression of *IL1B* and *IL1RN*. In contrast, greater *IL1RN* expression was seen in neutrophils in peripheral blood, compared to monocyte/macrophages. *IL‐6* expression was detected in monocyte/macrophages in hematoma fluid and peripheral blood, but not in neutrophils. Gene expression over time followed the same pattern as the relevant protein in each compartment, except for *IL1RN*, which increased in hematoma while the IL‐1Ra protein decreased.

Expression of *IL‐1B*, *IL‐6*, and *IL‐1RN* in monocyte/macrophages from hematoma fluid was significantly associated with respective protein concentrations (Table 2). *IL‐1RN* expression in peripheral blood monocyte/macrophages was associated with plasma IL‐1Ra. No association was found between gene expression in neutrophils and cytokine concentrations.

**TABLE 2:** Association between Monocyte/Macrophage and Neutrophil Gene Expression and Cytokine Concentrations in Hematoma and Plasma

Gene/protein	Cell type	Sample pairs	β‐Coefficient	95% CI	*p*
Hematoma fluid					
*IL‐1A*/IL‐1α	Monocyte	52	0.361	−0.170 to 0.892	0.182
	Neutrophil	46	−0.001	−0.589 to 0.586	0.996
*IL‐1B*/IL‐1β	Monocyte	52	0.455	0.224 to 0.686	<0.001
	Neutrophil	46	0.179	−0.104 to 0.463	0.215
*IL‐6*/IL‐6	Monocyte	52	0.200	0.012 to 0.388	0.004
	Neutrophil	–	–	–	–
*IL1RN*/IL‐1Ra	Monocyte	51	0.256	0.109 to 0.403	0.001
	Neutrophil	45	0.144	−0.017 to 0.305	0.08
Peripheral blood					
*IL‐6*/IL‐6	Monocyte	72	0.136	−0.132 to 0.405	0.319
	Neutrophil	–	–	–	–
*IL1RN*/IL‐1Ra	Monocyte	72	0.281	0.079 to 0.484	0.007
	Neutrophil	70	0.112	−0.055 to 0.278	0.189

Values are given as the β‐coefficient and 95% CI. *IL‐6* expression was undetectable in neutrophils. CI = confidence interval; IL = interleukin; IL‐1Ra = IL‐1 receptor antagonist.

Immunohistochemical reaction for IL‐1Ra on postmortem brains showed expression in a few neurons in perihematomal tissue at day 3 in 1 donor. Equivocal staining was seen in perilesional macrophages, and no staining was present in neutrophils and macrophages within the clot (Figure S1). Perihematomal neuronal IL‐1Ra expression was found postmortem on day 58 post‐ICH. IL‐1Ra was not present in neurons in controls or in other ICH cases. All ICH cases and the 3 controls demonstrated IL‐1Ra expression in occasional microglial cells and perivascular macrophages. Control tissue from colorectal carcinoma shows consistent expression in neutrophils and macrophages and staining of a few tumor cells.

### Association between Cytokine Concentrations in Hematoma and Plasma

Hematoma concentrations of IL‐1α, IL‐1β, IL‐6, and IL‐1Ra were compared to concentrations of IL‐6, CRP, and IL‐1Ra in plasma taken at the same time or later the same day (Table 3). Only hematoma IL‐1β was significantly associated with CRP (β‐coefficient: 20.9; 95% CI: 4.4–37.5).

**TABLE 3:** Association Between Blood and Brain Inflammatory Markers

Hematoma cytokines	Number of pairs, n	Plasma cytokines					
		IL‐6 (pg/mL, × 10^−3^)		CRP (mg/L)		IL‐1Ra (pg/mL, × 10^−3^)	
		β‐coefficient	95% CI	β‐coefficient	95% CI	β‐coefficient	95% CI
IL‐1α (pg/mL, × 10^−3^)	117	−0.051	−0.249 to 0.146	88.3	−30.5 to 207.1	−0.128	−2.791 to 2.535
IL‐1β (pg/mL, × 10^−3^)	117	0.011	−0.018 to 0.040	21.0*	4.4 to 37.5	0.120	−0.273 to 0.513
IL‐1Ra (pg/mL, × 10^−3^)	112	0.002	−0.001 to 0.006	0.077	−1.717 to 1.871	0.019	−0.021 to 0.060
IL‐6 (pg/mL, × 10^−3^)	117	0.0003	−0.0001 to 0.0006	0.0937	−0.118 to 0.305	0.003	−0.002 to 0.007

Values are given as the β‐coefficient and 95% CI. CI = confidence interval; CRP = C‐reactive protein; IL = interleukin; IL‐1Ra = IL‐1 receptor antagonist.

* *p* = 0.013.

### Plasma Cytokine Concentrations in Surgical versus Medical MISTIE III Trial Participants

**FIGURE 5:**
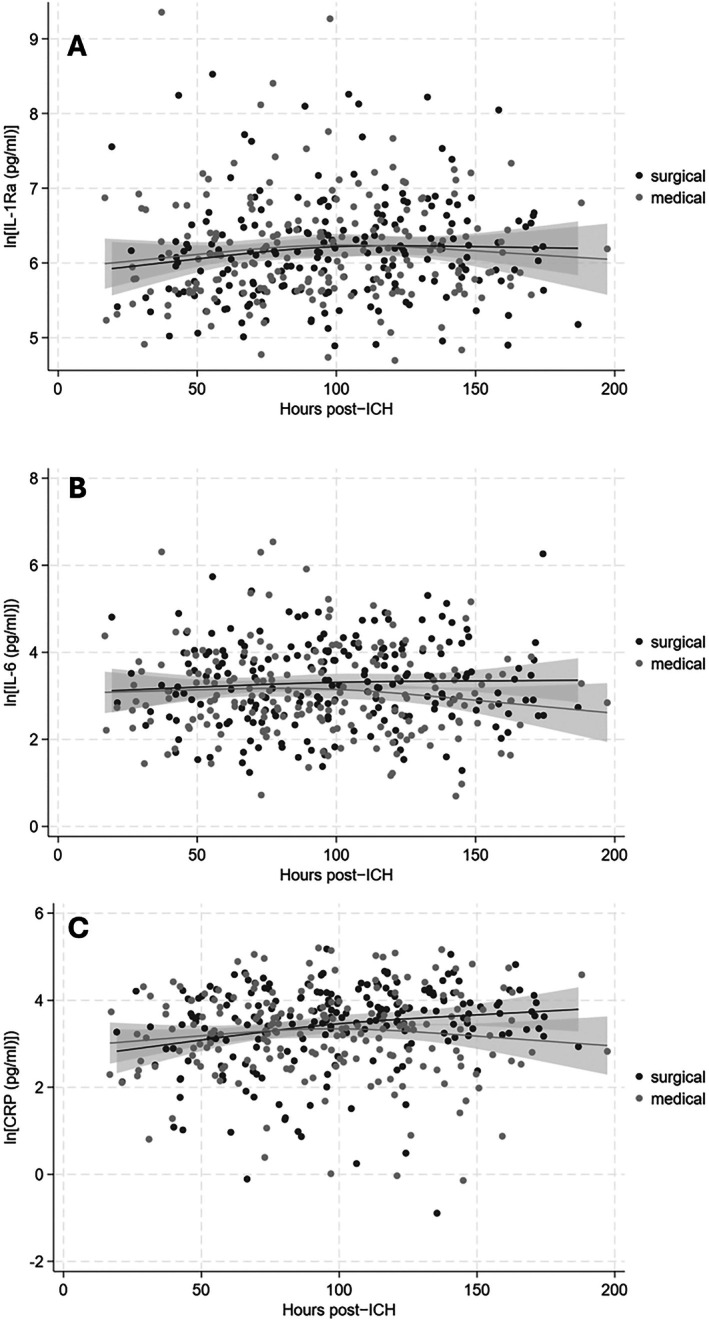
Time profile of circulating inflammatory markers by treatment group with patients randomized to surgical intervention in dark gray and those randomized to medical treatment only in light gray. Scatter plots are shown for each group against time from onset for (A) interleukin‐1 receptor antagonist (IL‐1Ra), (B) interleukin‐6 (IL‐6), and (C) C‐reactive protein (CRP). After adjusting for time from onset, severity index, and age, no difference was seen between medical and surgical groups for any of the 3 cytokines measured. An interaction term for time from onset and surgery indicated a significant interaction for IL‐6 and CRP, with both declining more slowly with time in the surgical group (vs medical).

Plasma IL‐6, CRP, and IL‐1Ra were compared by treatment group (surgical vs medical) (Fig 5). No significant difference was seen in IL‐6 (surgical vs medical; β‐coefficient: −0.26; 95% CI: −0.78 to 0.26), CRP (surgical vs medical; β‐coefficient: −0.21; 95% CI: −0.71 to 0.28), or IL‐1Ra (surgical vs medical; β‐coefficient: −0.11; 95% CI: −0.46 to 0.23), after adjusting for severity index, age, hours after onset, and with an interaction term for treatment group and hours after onset. There was a significant interaction between treatment group and hours after onset for IL‐6 and CRP, with *p*‐values of 0.02 and 0.03, respectively. There was no significant interaction for IL‐1Ra (*p*‐value for interaction = 0.44). IL‐6 and CRP appear to decline more over time in medical participants versus surgical participants (see Fig 5).

## Discussion

Using the unique opportunity provided by the MISTIE III trial, we have demonstrated that IL‐1, IL‐6, and IL‐1Ra are detectable in hematoma fluid in the first week after onset, in patients with ICH. Monocyte/macrophages within hematoma fluid seem to be a key source of IL‐1β, IL‐6, and IL‐1Ra. Higher concentrations of IL‐6 and IL‐1Ra in the hematoma were associated with good outcome at 1 year, IL‐1Ra was lower in lobar ICH (vs deep ICH). No cytokines in the hematoma or plasma were associated with PHE volume. Finally, higher hematoma IL‐1β was associated with higher plasma CRP and no significant difference was found in plasma cytokine concentrations in patients randomized to the MISTIE III surgical intervention versus medical care.

IL‐1 is a promising therapeutic target in stroke based on meta‐analyses of preclinical studies.^26^ Phase 2 trials have been conducted in ischemic stroke, subarachnoid hemorrhage, and ICH.^27^, ^28^, ^29^, ^30^, ^31^ The only completed trial of IL‐1Ra in ICH had day 3 edema extension distance as the primary outcome, but was underpowered because of the impact of the coronavirus disease 2019 pandemic on recruitment. Nevertheless, a significant reduction in plasma IL‐6 on day 2 was noted in a post hoc analysis. A further phase 2 trial of IL‐1Ra is underway and will provide further data.^32^

Our results advance the understanding of the therapeutic potential of IL‐1Ra. First, we have shown that the concentration of endogenous hematomal IL‐1Ra over the first week after ICH is independently associated with good outcome at 1 year. Second, we have shown that IL‐1 is present, but decreasing in the hematoma over the first week after onset so the treatment window for IL‐1Ra may be longer than the 72 hours tested in previous and ongoing trials.^31^, ^32^ Both ICH IL‐1Ra trial protocols include edema as the primary outcome, but we have found no association between IL‐1Ra in the hematoma and edema. Edema in our study may have been influenced from a complex interplay between the surgical intervention, the presence of alteplase in the tissue, and the endogenous inflammatory response. Alteplase was part of the MISTIE III surgical intervention and is known to have deleterious effects on neurons and glia.^33^ Finally, we have shown that IL‐1Ra is significantly lower in lobar hematomas. A recent positron emission tomography (PET) magnetic resonance (MR) study showed lobar ICH (vs deep) had more blood–brain barrier breakdown at day 1 to 3 using dynamic contrast enhanced MR imaging and more neuroinflammation from day 2 to 7 using PK‐11195 PET imaging.^23^ Another recent study using CT to monitor the evolution of edema in ICH patients to day 14 found PHE to increase from day 3 to 14 in lobar ICHs only.^34^ These findings may suggest a greater inflammatory response in lobar ICH, facilitated by low concentrations of IL‐1Ra, which may increase the potential benefit of treatment with IL‐1Ra in lobar ICH.

Our finding that higher hematoma IL‐6 concentrations are associated with a higher odds of a good outcome at one year was unexpected. IL‐6 has historically been considered a proinflammatory cytokine.^35^ For example, a recent study of cerebrospinal fluid (CSF) cytokines on admission with ICH showed CSF IL‐6 to be associated with worse outcome.^36^ Furthermore, IL‐6 in the plasma was shown to be associated with worse 90‐day mRS and larger ICH and PHE volumes.^37^ Early in the pathophysiology of ICH, infiltrating CCR2^+^ monocytes release IL‐6 and their depletion is associated with improved functional scores in rodents.^38^ However, other studies have identified the importance of IL‐6 at later time points. A study using rodent cerebrovascular injury models and human penumbral tissue around day 6 after onset, demonstrates that infiltrating CCR2^+^ monocytes act as a primary source for IL‐6, driving microglia to a phenotype with proliferative and proangiogenic properties, termed repair‐associated microglia, promoting functional repair after injury.^39^ It is also recognized that IL‐6 receptor α exists in a soluble and transmembrane form. When membrane‐bound, signaling via the IL‐6 receptor α induces anti‐inflammatory classic signaling, and the soluble IL‐6 receptor α induces pro‐inflammatory trans signaling, which may represent a key mechanism by which IL‐6 may drive both processes.^40^ Furthermore, IL‐6 may be required to promote early immune cell recruitment to the site of the ICH, which may be necessary for a later robust anti‐inflammatory response. In our study, IL‐6 was measured from day 1 to 2 up to day 7, but we did not identify any heterogeneity for the association between hematoma IL‐6 concentration and mRS at year. Whether the role of IL‐6 may be different as the inflammatory response evolves, initially promoting harmful inflammatory processes but later becoming essential for repair and recovery after ICH will need to be investigated in further studies.

Gene expression data from myeloid populations from just under half of INFLAME‐ICH surgical patients have shown that both monocyte/macrophages and neutrophils contribute to IL‐1 hematoma fluid protein concentrations. Monocyte/macrophages express more *IL1B* than neutrophils, and transcript expression for *IL1A* is similar in both cell types. Simple visual inspection suggests expression of *IL1RN* from both cell types in hematoma fluid appears to increase over time, contrary to decreasing IL‐1Ra concentration in hematoma fluid. Although this may be a chance finding attributable to gene expression data only being available for 50% of the patients, it may also be because of falling release from another *IL1RN* expressing cell. Although our postmortem brain analysis was very limited and exploratory, we have identified IL‐1Ra expression in a few neurons at the interface with the hematoma in an acute ICH patient who died on day 3, suggesting that neurons might also contribute IL‐1Ra. Perihematomal neuronal IL‐1Ra staining was also detected from an ICH patient who died at day 58, suggesting neuronal IL‐1Ra production may be prolonged.

We have observed *IL6* expression in monocyte/macrophages in both the hematoma fluid and plasma, but no detectable expression of *IL6* in neutrophils in either compartment. Through testing for correlations between gene expression and protein concentration, we have shown that gene expression of *IL‐1B*, *IL‐6*, and *IL‐1RN* in monocyte/macrophages contribute significantly to cytokine concentrations at the same time point in hematoma fluid, whereas neutrophils do not. In the peripheral blood, only *IL‐1RN* expression in monocyte/macrophages contributed significantly to plasma levels of IL‐1Ra.

No prior study has obtained serial hematoma and plasma samples over the first week in patients with ICH. Our study allowed us to show that concentrations of IL‐1β in the hematoma are associated with CRP in plasma samples collected on the same day. Importantly, this lends support to the use of plasma CRP as a surrogate measure of IL‐1β‐mediated inflammation in the hematoma of patients during the first week after ICH. It is of note that we found no association between IL‐1β and IL‐6, as IL‐6 is a well‐established mediator of CRP release from the liver. This may have arisen because of the higher variability of IL‐6 in the plasma of our sample (coefficient of variation of 1.62) when compared to CRP (0.81), making our analysis underpowered for the correlation between IL‐1β and IL‐6.

Neurosurgical interventions may promote neuroinflammation. We, therefore, tested whether randomization to surgery correlated to an increase in plasma inflammatory markers when compared to randomization to medical care. Although we found no significant increase in IL‐6, CRP, or IL‐1Ra with randomization to surgery, there was a significant interaction between treatment group and time from onset for IL‐6 and CRP, suggesting that both cytokines were decreasing more quickly in patients randomized to medical treatment. The MISTIE III intervention is minimally invasive and likely to cause much less tissue disruption and inflammation than conventional surgery. However, this finding suggests that the continued presence of the catheter in the hematoma is either directly or indirectly sustaining higher concentrations of IL‐6 and CRP over time. Whether this has any implications for outcomes is uncertain and will require further investigation.

The key strength of our study is the unique opportunity provided by MISTIE III to track inflammatory cytokines in hematoma and plasma of patients with ICH over the first week after onset. We have also benefited from the robust conduct of the MISTIE III trial. However, there remain some limitations. First, we have collected hematoma drain fluid, the composition of which may change over the time the drain remains in situ. The initial sample collected at the time of surgery is likely to be composed mostly of serum generated from the clotting of arterial blood and any unclotted blood. The contribution of perihematomal tissue is likely to increase over time, as the clot reduces in size with the MISTIE III intervention, and the duration of sample collection was dictated by the length of time the drain remained in situ, in turn dictated by the main trial protocol. Second, it remains possible that some of the drains implanted for INFLAME‐ICH patients were in communication with the ventricular space so that CSF was also present in the drain fluid, having a dilutional effect on cytokine concentrations and potentially introducing the same cytokines from the CSF compartment. Third, because of constraints on funding and the additional resource required to collect INFLAME‐ICH samples, we have only included a small proportion 87 of 506 (17%) of the MISTIE III trial participants from 21 of 78 (27%) study sites, reducing the power and possibly the generalizability of our findings. Our small sample size also limits our ability to adjust for all potential confounders in our regression models, but the use of the MISTIE III severity index in our regression models has allowed us to adjust for most key confounders. It is also important to note that we have undertaken multiple analyses without a prespecified analysis plan and some of the observed results could have arisen by chance. Our results must, therefore, be interpreted with caution and treated as exploratory. Fourth, to allow sufficient sample to accumulate for collection from hematoma drains, we allowed fluid to accumulate for 5 hours in the drip chamber. It is possible that cytokine concentrations were changing during this time. Pilot work before beginning the main study using *ex vivo* hematoma fluid samples from MISTIE III participants suggested that this was minimal and would not have been influenced by reducing the temperature to 4°C. Fifth, many of the samples collected from the hematoma were following administration of alteplase, as this was an integral part of the MISTIE III procedure. We are, therefore, unable to disentangle any effect of alteplase on the natural history of the inflammatory response. However, in 38 (81%) surgical patients, the first sample was collected at drain insertion, before administration of alteplase, and we note no significant differences in the concentration of hematoma cytokines measured in these samples when compared to first samples collected after alteplase administration in 9 surgical patients (Table S3), providing some reassurance that alteplase has not had a major effect on our results. The effects of alteplase on the immune response to stroke have been described previously in the literature. In animal models of ischemic stroke, alteplase has been shown to have non‐thrombolytic effects, including cleavage of platelet‐derived growth factor and activation of receptors including Annexin A2, low‐density lipoprotein receptor‐related protein 1, and the N‐methyl‐D‐aspartate receptor‐.^41^ This leads to activation of microglia and astrocytes, increasing the release of proinflammatory cytokines and other mediators. Activation of endothelial cells promotes matrix metalloproteinase 9 release, increasing blood–brain barrier permeability and cerebral edema.^41^ Much less is known about alteplase in ICH. In the Clot Lysis: Evaluation of Accelerated Resolution in Intraventricular Hemorrhage phase III (CLEAR III) trial, where patients with intraventricular hemorrhage (IVH) were randomized to receive alteplase or saline via an external ventricular drain, alteplase was shown to be associated with a significantly higher CSF white blood cell count (vs saline), even when adjusting for IVH volume and clearance rates.^42^ However, a non‐randomized study of 88 patients with IVH, 47 of whom received intraventricular alteplase, showed a trend to lower IL‐1β in the CSF of alteplase‐treated patients, which reached statistical significance at day 7.^34^ Therefore, there remains uncertainty about the role of alteplase in our findings, which may be addressed by studying samples from patients undergoing different types of minimally invasive surgery not requiring the use of alteplase. Finally, although 77 of 89 (87%) of participants had edema measured on a scan within a 24‐hour window of 120 hours +/− 12 hours, a minority of patients had edema measured outside this time, potentially introducing additional unwanted variation.

In conclusion, in our small exploratory analysis, we have demonstrated that IL‐1Ra in the hematoma of patients over the first week after ICH is independently associated with a favorable outcome at 1 year, providing some support for the further testing of IL‐1Ra and other IL‐1 inhibitors in ICH. The presence of hematoma IL‐1 up to day 7 and the rising concentrations of IL‐1Ra in patients with a good outcome suggest that the treatment window for IL‐1Ra might be longer than tested in completed and ongoing phase II trials. Finally, we have shown that higher hematoma IL‐6 is independently associated with a good outcome at 1 year, suggesting a possible role in the disease process for enhancing repair and recovery.

## Author Contributions

A.P.J., L.S., W.Z., M.A., and D.H. contributed to the conception and design of the study; A.P.J., B.N., M.G., M.A., M.N., Y.D., P.K., S.C., S.A., and F.R. contributed to the acquisition and analysis of data; A.P.J., B.N., M.N., Y.D., P.K., S.C., S.A., and F.R. contributed to drafting the text or preparing the figures.

## Potential Conflicts of Interest

Nothing to report.

## INFLAME‐ICH Investigators

Mark Harrigan*; David Miller; Judy Huang; Wendy Ziai*; Jack Jallo*; Fred Rincon; Gaurav Gupta; Igor Rybinnik*; Diederik Bulters*; Mary Leigh Gelea; Andras Buki*; Erzsebet Ezer; Hiren Patel*; Adrian Parry‐Jones; Charles Matouk; Kevin Sheth / Lauren Sansing*; Ali Zomorodi; Michael James*; Pal Barzo*; Krisztian Tanczos; Gregory Thompson; Ventatakrishna Rajajee/Aditya Pandey*; Peter Nakaji*; Shawn E. Wright; Walter Galicich; Thomas Bergman*; Andrew Carlson*; Huy Tran; Philipp Taussky; Safdar Ansari*; Fuat Arikan*; Marcelino Baguena; Ronald Reimer*; W. David Freeman; Ania Pollack; John Terry*; Geza Mezey; Katalin Szabo/Laszlo Csiba*; Robert F. James*; Steven J. Barrer; Larami MacKenzie*

## Supporting information


**Table S1.** Baseline characteristics of INFLAME‐ICH study patients compared to other MISTIE III participants. Show as median and interquartile range, where appropriate.
**Table S2:** Model fit for logistic regression models testing for an association between cytokine concentration and outcome at 1 year. Different models were tested, comparing no cut point for hematoma cytokine concentrations with varying cut points between 72 and 120 h post‐ICH.
**Table S3:** Comparison of hematoma cytokine concentrations in the first sample collected from each surgical patient by whether alteplase had been received or not. Concentrations between groups were compared using the Mann–Whitney *U* test.
**Figure S1:** The grey matter surrounding the hematoma of a donor who died three days after hemorrhagic stroke shows reactive gliosis and neurones with intensely eosinophilic cytoplasm and hyperchromatic nucleus indicating hypoperfusion (A, HE ‐ ×40); a few neurons show cytoplasmic staining for IL‐1Ra (B, immunoperoxidase – ×80); sparse microglial cells are also positive (arrow) (C, immunoperoxidase – ×80). Figure 1D represent the gliotic cortex adjacent to the hematoma in a donor who survived 58 days after stroke; a collection of hemosiderin‐laden macrophages is present (HE – ×40). Scattered neurones (arrow) (E, immunoperoxidase – ×80) and isolated microglial cells (arrow) (F, immunoperoxidase – ×80) stain for IL‐1Ra.

## Data Availability

INFLAME‐ICH data will be made available on reasonable request to the corresponding author. Contact The Johns Hopkins Brain Injury Outcomes (BIOS) Division for enquiries regarding access to MISTIE III data.

## References

[1] Wolsink A , Cliteur MP , van Asch CJ , et al. Incidence, case fatality, and functional outcome of intracerebral haemorrhage, according to age, sex, and country income level: a systematic review and meta‐analysis. Lancet Reg Health Eur 2025;49:101180. 39759582 10.1016/j.lanepe.2024.101180 PMC11699750

[2] Parry‐Jones AR , Krishnamurthi R , Ziai WC , et al. World stroke organization (WSO): global intracerebral hemorrhage factsheet 2025. Int J Stroke 2025;20:145–150. 39629687 10.1177/17474930241307876 PMC11786522

[3] Greenberg SM , Ziai WC , Cordonnier C , et al. 2022 guideline for the Management of Patients with Spontaneous Intracerebral Hemorrhage: a guideline from the American Heart Association/American Stroke Association. Stroke 2022;53:e282–e361. 35579034 10.1161/STR.0000000000000407

[4] Sondag L , Schreuder FHBM , Boogaarts HD , et al. Neurosurgical intervention for supratentorial intracerebral hemorrhage. Ann Neurol 2020;88:239–250. 10.1002/ana.25732. 32239722 PMC7497162

[5] Wilting FN , Sondag L , Schreuder FH , et al. Surgery for spontaneous supratentorial intracerebral haemorrhage. Cochrane Database Syst Rev 2025;7:CD015387. 40673401 10.1002/14651858.CD015387.pub2 PMC12269361

[6] Keep RF , Hua Y , Xi G . Intracerebral haemorrhage: mechanisms of injury and therapeutic targets. Lancet Neurol 2012;11:720–731. 22698888 10.1016/S1474-4422(12)70104-7 PMC3884550

[7] Schwartz M , Shechter R . Systemic inflammatory cells fight off neurodegenerative disease. Nat Rev Neurol 2010;6:405–410. 20531383 10.1038/nrneurol.2010.71

[8] Allan SM , Tyrrell PJ , Rothwell NJ . Interleukin‐1 and neuronal injury. Nat Rev Immunol 2005;5:629–640. 16034365 10.1038/nri1664

[9] Lu A , Tang Y , Ran R , et al. Brain genomics of intracerebral hemorrhage. J Cereb Blood Flow Metab 2006;26:230–252. 16034371 10.1038/sj.jcbfm.9600183

[10] Carmichael ST , Vespa PM , Saver JL , et al. Genomic profiles of damage and protection in human intracerebral hemorrhage. J Cereb Blood Flow Metab 2008;28:1860–1875. 18628781 10.1038/jcbfm.2008.77 PMC2745827

[11] Masada T , Hua Y , Xi G , et al. Overexpression of interleukin‐1 receptor antagonist reduces brain edema induced by intracerebral hemorrhage and thrombin. Acta Neurochir Suppl 2003;86:463–467. 14753487 10.1007/978-3-7091-0651-8_95

[12] O'Collins VE , Macleod MR , Donnan GA , et al. 1,026 experimental treatments in acute stroke. Ann Neurol 2006;59:467–477. 16453316 10.1002/ana.20741

[13] Castillo J , Davalos A , Alvarez‐Sabin J , et al. Molecular signatures of brain injury after intracerebral hemorrhage. Neurology 2002;58:624–629. 11865143 10.1212/wnl.58.4.624

[14] Alvarez‐Sabin J , Delgado P , Abilleira S , et al. Temporal profile of matrix metalloproteinases and their inhibitors after spontaneous intracerebral hemorrhage: relationship to clinical and radiological outcome. Stroke 2004;35:1316–1322. 15087562 10.1161/01.STR.0000126827.69286.90

[15] Leira R , Davalos A , Silva Y , et al. Early neurologic deterioration in intracerebral hemorrhage: predictors and associated factors. Neurology 2004;63:461–467. 15304576 10.1212/01.wnl.0000133204.81153.ac

[16] Silva Y , Leira R , Tejada J , et al. Molecular signatures of vascular injury are associated with early growth of intracerebral hemorrhage. Stroke 2005;36:86–91. 15550687 10.1161/01.STR.0000149615.51204.0b

[17] Di Napoli M , Godoy DA , Campi V , et al. C‐reactive protein in intracerebral hemorrhage: time course, tissue localization, and prognosis. Neurology 2012;79:690–699. 22855859 10.1212/WNL.0b013e318264e3be

[18] Castellanos M , Leira R , Tejada J , et al. Predictors of good outcome in medium to large spontaneous supratentorial intracerebral haemorrhages. J Neurol Neurosurg Psychiatry 2005;76:691–695. 15834028 10.1136/jnnp.2004.044347 PMC1739633

[19] Wu H , Zhang Z , Hu X , et al. Dynamic changes of inflammatory markers in brain after hemorrhagic stroke in humans: a postmortem study. Brain Res 2010;1342:111–117. 20420814 10.1016/j.brainres.2010.04.033 PMC2885522

[20] Hanley DF , Thompson RE , Rosenblum M , et al. Efficacy and safety of minimally invasive surgery with thrombolysis in intracerebral haemorrhage evacuation (MISTIE III): a randomised, controlled, open‐label, blinded endpoint phase 3 trial. Lancet 2019;393:1021–1032. 30739747 10.1016/S0140-6736(19)30195-3 PMC6894906

[21] Dinarello CA . Overview of the IL‐1 family in innate inflammation and acquired immunity. Immunol Rev 2018;281:8–27. 29247995 10.1111/imr.12621 PMC5756628

[22] Samarasekera N , Ferguson K , Parry‐Jones AR , et al. Perihaematomal Oedema evolution over 2 weeks after spontaneous intracerebral Haemorrhage and association with outcome: a prospective cohort study. Cerebrovasc Dis 2025;54:298–307. 38952101 10.1159/000540099

[23] Jones OA , Mohamed S , Hinz R , et al. Neuroinflammation and blood‐brain barrier breakdown in acute, clinical intracerebral hemorrhage. J Cereb Blood Flow Metab 2025;45:233–243. 39360420 10.1177/0271678X241274685 PMC11563506

[24] Mould WA , Carhuapoma JR , Muschelli J , et al. Minimally invasive surgery plus recombinant tissue‐type plasminogen activator for intracerebral hemorrhage evacuation decreases perihematomal edema. Stroke 2013;44:627–634. 23391763 10.1161/STROKEAHA.111.000411 PMC4124642

[25] Askenase MH , Goods BA , Beatty HE , et al. Longitudinal transcriptomics define the stages of myeloid activation in the living human brain after intracerebral hemorrhage. Sci Immunol 2021;6:eabd6279. 33891558 10.1126/sciimmunol.abd6279 PMC8252865

[26] Frantzias J , Sena ES , Macleod MR , Al‐Shahi Salman R . Treatment of intracerebral hemorrhage in animal models: meta‐analysis. Ann Neurol 2011;69:389–399. 21387381 10.1002/ana.22243

[27] Galea J , Ogungbenro K , Hulme S , et al. Reduction of inflammation after administration of interleukin‐1 receptor antagonist following aneurysmal subarachnoid hemorrhage: results of the subcutaneous interleukin‐1Ra in SAH (SCIL‐SAH) study. J Neurosurg 2018;128:515–523. 28298024 10.3171/2016.9.JNS16615

[28] Singh N , Hopkins SJ , Hulme S , et al. The effect of intravenous interleukin‐1 receptor antagonist on inflammatory mediators in cerebrospinal fluid after subarachnoid haemorrhage: a phase II randomised controlled trial. J Neuroinflammation 2014;11:1. 24383930 10.1186/1742-2094-11-1 PMC3892121

[29] Smith CJ , Hulme S , Vail A , et al. SCIL‐STROKE (subcutaneous Interleukin‐1 receptor antagonist in ischemic stroke): a randomized controlled phase 2 trial. Stroke 2018;49:1210–1216. 29567761 10.1161/STROKEAHA.118.020750

[30] Emsley HCA , Smith CJ , Georgiou RF , et al. A randomised phase II study of interleukin‐1 receptor antagonist in acute stroke patients. J Neurol Neurosurg Psychiatry 2005;76:1366–1372. 16170078 10.1136/jnnp.2004.054882 PMC1739363

[31] Parry‐Jones AR , Stocking K , MJ ML , et al. Phase II randomised, placebo‐controlled, clinical trial of interleukin‐1 receptor antagonist in intracerebral haemorrhage: BLOcking the cytokine IL‐1 in ICH (BLOC‐ICH). Eur Stroke J 2023;8:819–827. 37452707 10.1177/23969873231185208 PMC10472954

[32] Cliteur M , van der Kolk A , Hannink G , et al. Anakinra in cerebral haemorrhage to target secondary injury resulting from neuroinflammation (ACTION): study protocol of a phase II randomised clinical trial. Eur Stroke J 2024;9:265–273. 37713268 10.1177/23969873231200686 PMC10916813

[33] Benchenane K , López‐Atalaya JP , Fernández‐Monreal M , et al. Equivocal roles of tissue‐type plasminogen activator in stroke‐induced injury. Trends Neurosci 2004;27:155–160. 15036881 10.1016/j.tins.2003.12.011

[34] Samarasekera N , Ferguson K , Parry‐Jones AR , et al. Perihaematomal Oedema evolution over 2 weeks after spontaneous intracerebral Haemorrhage and association with outcome: a prospective cohort study. Cerebrovasc Dis 2025;54:298–307. 38952101 10.1159/000540099

[35] Taylor RA , Chang C‐F , Goods BA , et al. TGF‐β1 modulates microglial phenotype and promotes recovery after intracerebral hemorrhage. J Clin Invest 2017;127:280–292. 27893460 10.1172/JCI88647 PMC5199690

[36] Gu T , Pan J , Chen L , et al. Association of inflammatory cytokines expression in cerebrospinal fluid with the severity and prognosis of spontaneous intracerebral hemorrhage. BMC Neurol 2024;24:7. 38167007 10.1186/s12883-023-03487-x PMC10759732

[37] Leasure AC , Kuohn LR , Vanent KN , et al. Association of Serum IL‐6 (interleukin 6) with functional outcome after intracerebral hemorrhage. Stroke 2021;52:1733–1740. 33682454 10.1161/STROKEAHA.120.032888 PMC8085132

[38] Hammond MD , Taylor RA , Mullen MT , et al. CCR2+ Ly6C(hi) inflammatory monocyte recruitment exacerbates acute disability following intracerebral hemorrhage. J Neurosci 2014;34:3901–3909. 24623768 10.1523/JNEUROSCI.4070-13.2014 PMC3951693

[39] Choi B‐R , Johnson KR , Maric D , DB MG . Monocyte‐derived IL‐6 programs microglia to rebuild damaged brain vasculature. Nat Immunol 2023;24:1110–1123. 37248420 10.1038/s41590-023-01521-1 PMC11531796

[40] Reeh H , Rudolph N , Billing U , et al. Response to IL‐6 trans‐ and IL‐6 classic signalling is determined by the ratio of the IL‐6 receptor α to gp130 expression: fusing experimental insights and dynamic modelling. Cell Commun a Signal 2019;17:46. 10.1186/s12964-019-0356-0 PMC6525395 31101051

[41] Liu Q , Shi K , Wang Y , Shi F‐D . Neurovascular inflammation and complications of thrombolysis therapy in stroke. Stroke 2023;54:2688–2697. 37675612 10.1161/STROKEAHA.123.044123

[42] Fam MD , Zeineddine HA , Eliyas JK , et al. CSF inflammatory response after intraventricular hemorrhage. Neurology 2017;89:1553–1560. 28887375 10.1212/WNL.0000000000004493 PMC5634667

