# Supplementary material for: Hematoma Interleukin‐1 Receptor Antagonist Concentrations Predict Long‐Term Outcome in Acute Human Intracerebral Hemorrhage
Source: Ann Neurol. 2026 May 3;100(2):345–60. doi: 10.1002/ana.78237 (PMC13387941; doi:10.1002/ana.78237)
Supplement: Supplementary file 1 — Table S1. Baseline characteristics of INFLAME‐ICH study patients compared to other MISTIE III participants. Show as median and interquartile range, where appropriate. Table S2: Model fit for logistic regression models testing for an association between cytokine concentration and outcome at 1 year. Different models were tested, comparing no cut point for hematoma cytokine concentrations with varying cut points between 72 and 120 h post‐ICH. Table S3: Comparison of hematoma cytokine concentrations in the first sample collected from each surgical patient by whether alteplase had been received or not. Concentrations between groups were compared using the Mann–Whitney U test. Figure S1: The grey matter surrounding the hematoma of a donor who died three days after hemorrhagic stroke shows reactive gliosis and neurones with intensely eosinophilic cytoplasm and hyperchromatic nucleus indicating hypoperfusion (A, HE ‐ ×40); a few neurons show cytoplasmic staining for IL‐1Ra (B, immunoperoxidase – ×80); sparse microglial cells are also positive (arrow) (C, immunoperoxidase – ×80). Figure 1D represent the gliotic cortex adjacent to the hematoma in a donor who survived 58 days after stroke; a collection of hemosiderin‐laden macrophages is present (HE – ×40). Scattered neurones (arrow) (E, immunoperoxidase – ×80) and isolated microglial cells (arrow) (F, immunoperoxidase – ×80) stain for IL‐1Ra. [file ANA-100-345-s001.docx]

**Supplementary methods:**

*MISTIE-III Severity index*

The MISTIE-III severity index was derived and described for the MISTIE-III trial and is calculated as follows:

Severity Index = 1.9*(1 if age <56; 0 otherwise) + 1.2*(1 if age ≥56 and <67; 0 otherwise) + 0*(1 if age ≥67; 0 otherwise) + 0.8*(1 if GCS 13-15; 0 otherwise) + 0.6*(1 if GCS 9–12; 0 otherwise) + 0*(1 if GCS 3–8; 0 otherwise) + 1.9*(1 if ICH location lobar; 0 otherwise) +0*(1 if ICH location deep; 0 otherwise) + 0.9*(1 if stability ICH <45 mL; 0 otherwise) + 0*(1 if stability ICH ≥45 mL; 0 otherwise) + 0.7*(1 if stability IVH ≤0.4 mL; 0 otherwise) + 0*(1 if stability IVH >0.4 mL; 0 otherwise) + 0.9*(1 if diabetes no; 0 otherwise) + 0*(1 if diabetes yes; 0 otherwise) + 1.2*(1 if Fazekas total score ≤ 2; 0 otherwise) + 0.6*(1 if Fazekas total score = 3; 0 otherwise ) + 0*(1 if Fazekas total score ≥4; 0 otherwise) . This severity score gives a maximum possible value of 8.3, with the highest score seen in the data at this maximum value.

*Postmortem brain tissue analysis*

Postmortem brain tissue was collected via the Edinburgh Brain Bank (ethics approval from East of Scotland Research Ethics Service, 21/ES/0087) in line with the Human Tissue (Scotland) Act 2006. The use of human tissue for post-mortem studies has been reviewed and approved by the Edinburgh Brain Bank ethics committee. Formalin-fixed and paraffin embedded (FFPE) samples for this study were selected from brain tissue immediately adjacent to the hematoma from three donors who died in the acute stage after ICH at day 2 (one case) and day 3, and three donors who died later after ICH, at days 41, 58 and 161. Control brain tissue was obtained from three donors without neurological conditions. Six samples were taken from the basal ganglia (three donors with ICH and three controls) and three samples include cortex and subcortical white matter. Post-mortem delay was available in five instances and ranged between 24 and 96 h (average 52 h). Sections of colorectal carcinoma was used as positive control.

The sections were deparaffinised in xylene and rehydrated in decreasing alcohols to distilled water. One section was stained with haematoxylin-eosin (H&E). For peroxidase immunohistochemistry, antigen unmasking was performed with Heat-Induced Epitope Retrieval method using 10mM Citrate buffer pH 6 in a steamer for 20 min. Endogenous peroxidase activity was quenched using 0.3% hydrogen peroxide in methanol for 30 min at room temperature. The sections were then incubated with normal blocking serum for 30 min at room temperature, followed by 60 min incubation at room temperature with anti-IL-1Ra polyclonal antibody recognising the three isoforms (Rabbit, Proteintech, 10844-1-AP) at 1:300 dilution. Biotinylated secondary antibody and peroxidase-linked Avidin Biotin Complex as per Vectastain Elite ABC-HRP kit (Vector Labs, PK-6101), and DAB substrate as per Vector DAB Peroxidase substrate kit (Vector Labs, SK-4100). After the reaction was developed, nuclear counterstaining was performed using Meyer’s haematoxylin, after which the sections were dehydrated in progressive alcohols to xylene, and coverslipped. The H&E-stained sections and the sections stained for IL-1Ra were scanned at x40 magnification with a Hamamatsu NanoZoomer S360 brightfield digital scanner (Welwyn Garden City, Hertfordshire, UK).

**Supplementary Table 1: Baseline characteristics of INFLAME-ICH study patients compared to other MISTIE-III participants.** Show as median and interquartile range, where appropriate.

|  | **INFLAME-ICH participants (n=89)** | **MISTIE-III participants not included in INFLAME-ICH (n=417)** | **All MISTIE-III participants (n=506)** |
| --- | --- | --- | --- |
| Age | 63 (54 to 70) | 62 (52 to 71) | 62 (52 to 71) |
| Sex, Male | 58 (65.2%) | 251 (60.2%) | 309 (61.1%) |
| Race  African American  Asian  White  Other | 13 (14.6%)  4 (4.5%)  71 (79.8%)  1 (1.1%) | 76 (18.2%)  26 (6.2%)  308 (74%)  7 (1.6%) | 89 (17.6%)  30 (5.9%)  379 (75%)  8 (1.5%) |
| On anticoagulants | 7 (7.9%) | 28 (6.7%) | 35 (6.9%) |
| Diabetes | 17 (19.1%) | 124 (29.7%) | 141 (27.9%) |
| Hypertension | 83 (93.3%) | 405 (97.1%) | 488 (96.4%) |
| Glasgow Coma Scale at randomisation | 11 (9 to 13) | 10 (8 to 13) | 10 (8 to 13) |
| NIHSS score at randomisation | 18 (15 to 22) | 19 (15 to 24) | 19 (15 to 23) |
| Severity index | 3.4 (2.1 to 4.1) | 3 (2.2 to 3.9) | 3.2 (2.2 to 4.1) |
| ICH location  Deep  Lobar | 51 (57.3%)  38 (42.7%) | 259 (62.1%)  158 (37.9%) | 310 (61.3%)  196 (38.7%) |
| ICH volume at baseline (mL) | 43.9 (33.5 to 57.1) | 41.5 (29.9 to 54.3) | 41.8 (30.7 to 54.4) |
| IVH volume at stability (mL) | 0.0 (0.0 to 2.0) | 0.5 (0 to 3.4) | 0.4 (0 to 3.2) |
| Ventilated at randomisation | 31 (34.8%) | 181 (43.4%) | 212 (41.9%) |

**Supplementary Table 2:** Model fit for logistic regression models testing for an association between cytokine concentration and outcome at 1 year. Different models were tested, comparing no cut point for hematoma cytokine concentrations with varying cut points between 72 h and 120 h post-ICH.

| **Cytokine** | **Cut point (h)** | **Included cases** | **AIC** | **BIC** |
| --- | --- | --- | --- | --- |
| **IL-1α** | None | 45 | 61.25 | 73.89 |
|  | 72 | 31 | 40.06 | 51.53 |
|  | 84 | 38 | 47.82 | 60.92 |
|  | 96 | 34 | 47.46 | 59.67 |
|  | 108 | 33 | 46.34 | 58.31 |
|  | 120 | 19 | 0 | 0 |
| **IL-1β** | None | 45 | 59.60 | 72.25 |
|  | 72 | 31 | 43.92 | 55.39 |
|  | 84 | 38 | 51.34 | 64.44 |
|  | 96 | 34 | 50.75 | 62.96 |
|  | 108 | 33 | 42.79 | 54.76 |
|  | 120 | 19 | 0.00 | 0.00 |
| **IL-1Ra** | None | 38 | 46.27 | 57.73 |
|  | 72 | 26 | 34.25 | 44.32 |
|  | 84 | 31 | 37.99 | 49.47 |
|  | 96 | 28 | 39.21 | 49.87 |
|  | 108 | 28 | 34.30 | 44.95 |
|  | 120 | 16 | 0.00 | 0.00 |
| **IL-6** | None | 45 | 49.90 | 62.55 |
|  | 72 | 31 | 39.48 | 50.96 |
|  | 84 | 38 | 48.97 | 62.07 |
|  | 96 | 34 | 48.76 | 60.97 |
|  | 108 | 33 | 44.30 | 56.28 |
|  | 120 | 19 | 26.74 | 33.35 |

**Supplementary table 3:** comparison of hematoma cytokine concentrations in the first sample collected from each surgical patient by whether alteplase had been received or not. Concentrations between groups were compared using the Mann-Whitney U test.

|  | First sample before alteplase exposure (n=38; median, IQR) | First sample after alteplase exposure  (n=9; median, IQR) | *p*-value |
| --- | --- | --- | --- |
| IL-1α (ng/ml) | 2.5 (0.2 to 10.7) | 5.5 (1.2 to 20.8) | 0.12 |
| IL-1β (ng/ml) | 20.0 (5.2 to 43.2) | 25.6 (4.7 to 67.7) | 0.77 |
| IL-1Ra (ng/ml) | 3697.0 (1829.0 to 6572.0) | 4983.5 (2919.0 to 6750.0) | 0.48 |
| IL-6 (ng/ml) | 15698.0 (5385.3 to 38319.1) | 25662.8 (4744.7 to 37196.8) | 0.76 |
| Onset to sample collection (h) | 83.1 (67.0 to 115.9) | 95.3 (75.7 - 131.0) |  |

**Supplementary Figure**: The grey matter surrounding the hematoma of a donor who died three days after hemorrhagic stroke shows reactive gliosis and neurones with intensely eosinophilic cytoplasm and hyperchromatic nucleus indicating hypoperfusion (A, HE - x40); a few neurons show cytoplasmic staining for IL-1Ra (B, immunoperoxidase – x80); sparse microglial cells are also positive (arrow) (C, immunoperoxidase – x80). Figure 1D represent the gliotic cortex adjacent to the hematoma in a donor who survived 58 days after stroke; a collection of hemosiderin-laden macrophages is present (HE – x40). Scattered neurones (arrow) (E, immunoperoxidase – x80) and isolated microglial cells (arrow) (F, immunoperoxidase – x80) stain for IL-1Ra.

**INFLAME-ICH Investigators** (in order of highest enrolment)

*Principial Investigator

| **Site Name** | **Lead Neurosurgeon** | **Neurointensivist/Other Principal Investigator** | **Study Coordinator** | **Number enrolled** |
| --- | --- | --- | --- | --- |
| University of Alabama at Birmingham | Mark Harrigan* | David Miller | Lisa Nelson | 14 |
| Johns Hopkins University | Judy Huang | Wendy Ziai* | Mirinda Anderson White | 9 |
| Thomas Jefferson University Hospital | Jack Jallo* | Fred Rincon | Laura Boyden and Jaime Dougherty | 8 |
| Rutgers University | Gaurav Gupta | Igor Rybinnik* | Michelle Moccio | 8 |
| University of Southampton | Diederik Bulters* | Mary Leigh Gelea | Jisha Jacob | 8 |
| University of Pécs | Andras Buki* | Erzsebet Ezer | Péter Csécsei | 6 |
| Salford Royal Hospital | Hiren Patel* | Adrian Parry-Jones | Victoria O’Loughlin | 5 |
| Yale University | Charles Matouk | Kevin Sheth / Lauren Sansing* | Kimberly Kunze, David Mampre, Sara Jasak | 5 |
| Duke University Medical Center | Ali Zomorodi | Michael James* | Erlinda Yeh | 4 |
| University of Szeged | Pal Barzo* | Krisztian Tanczos | Eniko Fako | 4 |
| University of Michigan | Gregory Thompson | Ventatakrishna Rajajee/Aditya Pandey* | Ron Ball | 3 |
| Barrow Neurological Institute | Peter Nakaji* | Shawn E. Wright | Norissa Honea | 2 |
| Hennepin County Medical Center | Walter Galicich | Thomas Bergman* | Kathryn France | 2 |
| University of New Mexico | Andrew Carlson* | Huy Tran | Amal Alchbli | 2 |
| University of Utah Hospital | Philipp Taussky | Safdar Ansari* | Crystal Neate, Julie Kay Martinez, Joshua Letsinger, Lilly Fagatele, Carol Eaquinto | 2 |
| Hospital Universitari Vall d'Hebron, Barcelona | Fuat Arikan* | Marcelino Baguena | Mireia Sanchis | 2 |
| Mayo Clinic, Jacksonville | Ronald Reimer* | W. David Freeman | Cristin Williams, Emily Edwards | 1 |
| Miami Valley Hospital | Ania Pollack | John Terry* | Angela Shoen | 1 |
| University of Debrecen | Geza Mezey | Katalin Szabo/Laszlo Csiba* | Katalin Szabo | 1 |
| University of Louisville | Robert F. James* |  | Ann Jerde | 1 |
| Abington Hospital – Jefferson Health | Steven J. Barrer | Larami MacKenzie* | Karin Jonczak and Patricia Bussinger | 1 |
